# Review of the Neural Oscillations Underlying Meditation

**DOI:** 10.3389/fnins.2018.00178

**Published:** 2018-03-26

**Authors:** Darrin J. Lee, Edwin Kulubya, Philippe Goldin, Amir Goodarzi, Fady Girgis

**Affiliations:** ^1^Neurosurgery, University of Toronto, Toronto, ON, Canada; ^2^Neurosurgery, University of California, Davis, Davis, CA, United States; ^3^Nursing, University of California, Davis, Davis, CA, United States

**Keywords:** meditation, EEG, MEG, focused attention, open-monitoring, transcendental

## Abstract

**Objective:** Meditation is one type of mental training that has been shown to produce many cognitive benefits. Meditation practice is associated with improvement in concentration and reduction of stress, depression, and anxiety symptoms. Furthermore, different forms of meditation training are now being used as interventions for a variety of psychological and somatic illnesses. These benefits are thought to occur as a result of neurophysiologic changes. The most commonly studied specific meditation practices are focused attention (FA), open-monitoring (OM), as well as transcendental meditation (TM), and loving-kindness (LK) meditation. In this review, we compare the neural oscillatory patterns during these forms of meditation.

**Method:** We performed a systematic review of neural oscillations during FA, OM, TM, and LK meditation practices, comparing meditators to meditation-naïve adults.

**Results:** FA, OM, TM, and LK meditation are associated with global increases in oscillatory activity in meditators compared to meditation-naïve adults, with larger changes occurring as the length of meditation training increases. While FA and OM are related to increases in anterior theta activity, only FA is associated with changes in posterior theta oscillations. Alpha activity increases in posterior brain regions during both FA and OM. In anterior regions, FA shows a bilateral increase in alpha power, while OM shows a decrease only in left-sided power. Gamma activity in these meditation practices is similar in frontal regions, but increases are variable in parietal and occipital regions.

**Conclusions:** The current literature suggests distinct differences in neural oscillatory activity among FA, OM, TM, and LK meditation practices. Further characterizing these oscillatory changes may better elucidate the cognitive and therapeutic effects of specific meditation practices, and potentially lead to the development of novel neuromodulation targets to take advantage of their benefits.

## Introduction

Meditation practices have well-established benefits in affective and cognitive processes (Tang et al., 2015). However, there are a wide variety of meditation practices, which comprise a set of practices used to cultivate positive qualities in the mind and to enhance insight into how the mind-body functions. Furthermore, the definition of meditation has many different meanings in varying contexts. While Christian, Judaic, and Islamic forms of meditation are generally devotional or scriptural, other forms of meditation are aimed at internally self-regulating the mind. It has been proposed that cognitive and affective effects may differ according to the type of meditation performed (Lutz et al., 2008a,b). This review focuses on four common meditation practices including focused attention (FA), open-monitoring (OM), transcendental meditation (TM), and loving-kindness (LK).

Two commonly studied types of attention training practices include FA and OM. Focused attention (FA) includes Himalayan Yoga, Mantra, and Metta; while OM includes Zen, Isha Yoga, Shoonya Yoga, and Vipassana. FA and OM have been shown to enhance attention control, emotion regulation, self-awareness, and improve cognitive control of conflict (Lippelt et al., 2014; Tang et al., 2015). FA is the practice of maintaining a sustained selective attention on a chosen concept or object, such as breathing, physical sensation, or a visual image. The chosen object serves as an anchor for attention, and as a result, FA is thought to cultivate mental acuity and focus. OM, on the other hand, entails focusing on awareness itself. Instead of sustaining selective attention on a chosen object and avoiding intrusive thoughts or distractions, OM involves acceptance of internal and external cues with the goal of non-judgmental awareness. The aim of OM is to remain attentive to any experience that may arise without focusing on a particular object. Of note, OM and FA are not mutually exclusive, and while OM practices may entail some aspects of FA, the opposite is not necessarily true. For example, OM practices often start by focusing on a specific object, similar to FA practices. Rather than maintaining that focus as the primary goal, as is the case in FA, the mind in OM is trained to gradually shift focus from that object and become aware of the occurrence of thoughts, sensations, and images as they arise and dissolve, and eventually begin monitoring the process of awareness itself. Therefore, OM induces a broader attentional focus than FA (Lippelt et al., 2014), and OM practitioners tend to have a generally wider attentional scope and perform better on sustained attention tasks (Ainsworth et al., 2013; Lippelt et al., 2014).

Transcendental meditation (TM) is a mantra meditation aimed at avoiding distracting thoughts. The goal is to use a sound or mantra to be aware of the present without an object of thought. In this practice, there is no contemplation, FA, or monitoring of current experience (Travis and Pearson, 2000). In contrast, LK meditation aims to develop love and compassion for oneself and toward all other beings. The meditator will eventually focus on compassion toward those one does not know and extend it toward those one dislikes. Negative associations are replaced with positive social or empathic mindsets (Vago and Silbersweig, 2012; Lippelt et al., 2014). As such, LK meditation has been associated with improved cognitive control and conflict monitoring (Hunsinger et al., 2013).

While the benefits of FA, OM, TM, and LK meditation have been described elsewhere, the neurobiological underpinnings of these benefits are still in its nascent phase. There is evidence that these forms of meditation result in both long-term and short-term changes in the brain. Long-term anatomical changes have traditionally been analyzed with structural magnetic resonance (MR) imaging, which provides excellent spatial resolution. There is evidence that meditation can result in structural changes in the brain, including increasing the cortical thickness of regions like the prefrontal cortex (PFC) and insula (Lazar et al., 2005; Santarnecchi et al., 2014; Engen et al., 2017). Additionally, functional MR imaging can detect changes in cortical and subcortical activation as well as functional connectivity; however, the temporal resolution for such changes are limited. In contrast, electroencephalography (EEG), magnetoencephalography (MEG), and source-space EEG are imaging modalities with excellent temporal resolution that can capture short-term oscillatory changes during meditation while sacrificing spatial resolution. Understanding the oscillations associated with different forms of meditation will aid in the honing of these meditative practices, and potentially allow for artificial manipulation in the treatment of disease.

In this review, we systematically analyze the similarities and differences in neural oscillations among four commonly studied meditation practices, including FA, OM, TM, and LK meditation.

## Neuronal activation during meditation

Functional MRI (fMRI) studies have demonstrated that various types of meditation increase activity in various regions of the brain, including the PFC, insula, and anterior cingulate cortex (ACC). Interestingly, different forms of meditation can activate different regions of the brain. FA results in increased brain activity and connectivity in the ACC relative to OM (Lazar et al., 2000; Botvinick et al., 2004; Manna et al., 2010). FA is also associated with increased right dorsolateral PFC activity and connectivity to the right insula, which has not been seen in OM (D'Esposito, 2007). In addition, both FA and OM demonstrate increased fMRI signal in the posterior insula during interoceptive attention tasks when compared to exteroception tasks (Farb et al., 2013). As can be expected, these forms of meditation are also associated with increased connectivity in brain networks such as the dorsal attentional network (Froeliger et al., 2012).

In contrast, most meditation practices, including FA, OM, and LK, are thought to deactivate the default mode network (DMN). This DMN is active during passive awake rest or involuntary activities and includes the ventral medial PFC, medial temporal lobe, precuneus, and posterior cingulate gyrus (Brewer et al., 2011; Garrison et al., 2015; Simon and Engstrom, 2015). Of note, TM results in continued elevation in DMN activity (Travis and Parim, 2017).

While it is clear that meditation practices can activate specific brain regions and functional connectivity associated with executive function and mood, as measured through fMRI, these practices also affect the neural oscillation patterns in these regions. In particular, neural oscillations can be evaluated in a local region or between various regions of the brain. The strength of a particular oscillatory frequency in a particular region can be analyzed using a power analysis. Coherence is the degree of coupling of a particular frequency between two different brain regions and can be used as an indicator of functional connectivity. EEG and MEG can both be used to investigate power and coherence within a particular frequency band. Understanding how meditation modulates these neural oscillations may help elucidate the relationship between brain oscillations and cognitive processes.

## Delta frequency

Delta oscillations arise from the thalamus or cortex and range between 0.5 and 3 Hz. In the context of meditation and neural oscillations, the role of delta frequency is not well-described. There is limited evidence suggesting that the delta frequency is reduced during OM, such as the Vipassana tradition. In one long-term Vipassana study, bilateral frontal delta power (1–4 Hz), but not midline delta power, was decreased in those who reported not being drowsy during meditation (Cahn et al., 2010). Of note, increased slow delta activity during deep sleep and increased delta activity during meditation suggests that the changes in delta during meditation promote an enhanced state of wakefulness. Similarly, LK meditation has been associated with increased delta activity (Basar et al., 2008). Interestingly, in a separate study, meditation evinced a reduction in delta activity; however, when a distractor item was presented and the meditator was encouraged to focus on the distractor item, the frontal delta power increased (Cahn et al., 2013). This suggests a potential role of the delta rhythm in attentional engagement.

## Theta frequency

The human theta rhythm is an oscillatory pattern found in cortical and subcortical structures, characterized by oscillations in the 3.5–7 Hz range. In humans, increased cortical theta oscillations have been described during a variety of learning tasks, including recognition (Raghavachari et al., 2001; Hsieh et al., 2011), recall (Sederberg et al., 2003), and virtual spatial navigation tasks (Kahana et al., 1999; de Araujo et al., 2002; Caplan et al., 2003; Watrous et al., 2011). In addition to local oscillatory activity, theta rhythms are synchronized across multiple brain regions during complex cognitive tasks (Mizuhara et al., 2004; Ekstrom et al., 2005). Increased cortical theta activity has also been demonstrated during working memory tasks (Raghavachari et al., 2001, 2006). Scalp EEG studies have demonstrated that increased theta activity prior to a memory task is correlated with successful episodic memory retrieval, while decreased theta activity has been associated with poor episodic memory (Addante et al., 2011). While meditation is known to improve attention, learning, and memory (Chan et al., 2017; Taren et al., 2017), there have been no causal studies on the role of meditation EEG changes and memory. EEG neurofeedback studies suggest improvements in cognition, including attention, procedural memory, and recognition memory (Gruzelier, 2014). Integrating neurofeedback into meditation may help to better define the relationship between memory and meditation.

**Figure 1:**
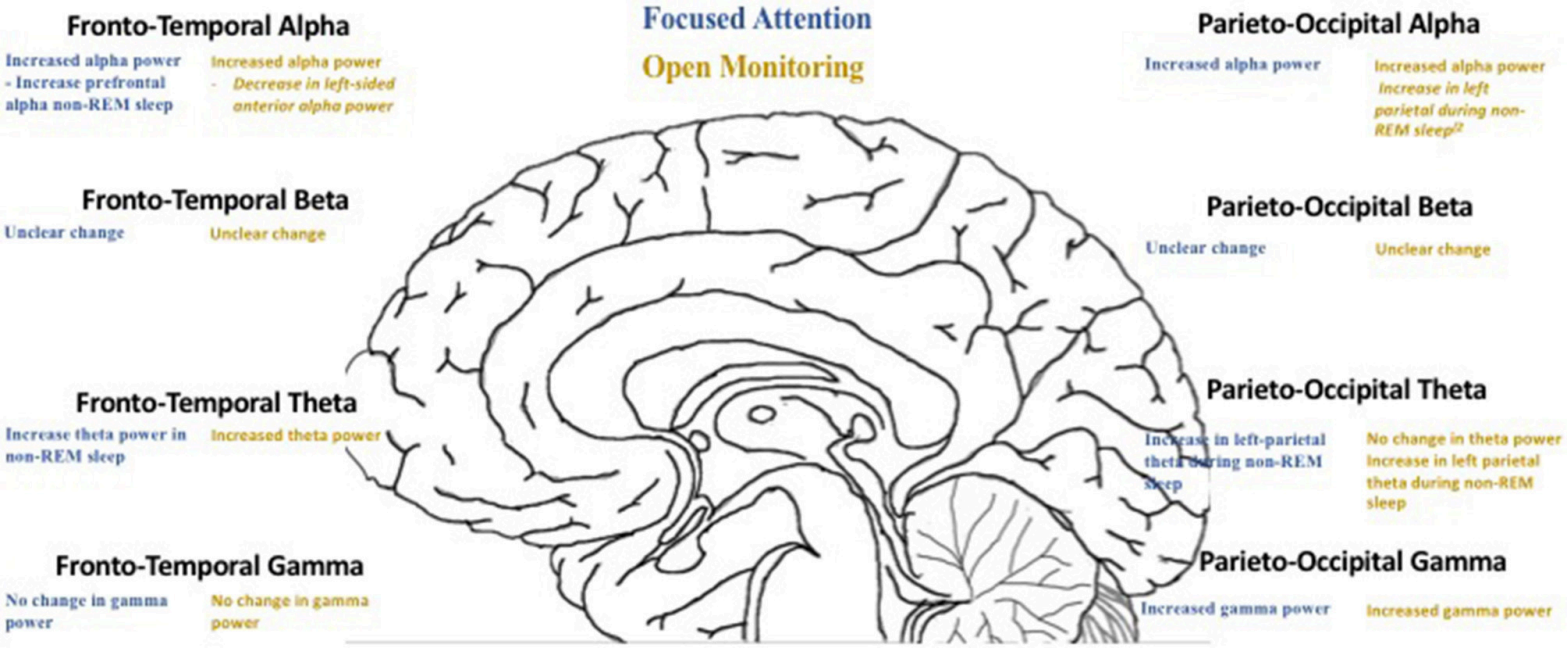
EEG oscillation contrasts between focused attention and open monitoring meditation practices.

Increases in theta activity have been seen across a variety of meditation practices, including FA, OM, TM, and LK (Baijal and Srinivasan, 2010; Cahn et al., 2010; Pasquini et al., 2015). Interestingly, the increase in theta power was positively correlated with the amount of training and experience in each meditation practice, which may help to explain the improvements in memory and attention. Theta oscillations during wakefulness occur in frontal midline regions, such as the PFC (Asada et al., 1999) and ACC (Onton et al., 2005) (Figure 1). This frontal midline theta (Fm theta) activity has been associated with concentrative attentional engagement (Basar et al., 2001; Mitchell et al., 2008) as well as activation of the autonomic system (Kubota et al., 2001; Takahashi et al., 2005). In particular, there is evidence for increases in Fm theta in both FA and OM (Takahashi et al., 2005; Dentico et al., 2016; Braboszcz et al., 2017). Fm theta is thought to be associated with internalized attention. As such, there is increased Fm theta during OM practice (Lippelt et al., 2014). Fm theta activity in Zen meditators was correlated with increased parasympathetic activity and correlated with decreased sympathetic activation, supporting the idea of the ACC as a source of Fm theta given its role in cognitive function and autonomic control.

In addition to the amplitude of the theta frequency, there are also changes in theta coherence (i.e., synchrony of neural firing patterns) during OM meditation. While frontal and parietal theta coherence is associated with executive function tasks such as working memory (Sauseng et al., 2005), similar findings have been shown during OM (Cahn et al., 2013). There is also evidence for increased theta coherence among the central, temporal and occipital areas during TM (Tomljenovic et al., 2016). However, this has not been investigated in FA meditation.

## Alpha frequency

The alpha frequency band ranges between 8 and 13 Hz, is predominantly in the occipital cortex, and is most notably seen in various stages of sleep. Both FA and OM meditation have been associated with increases in prefrontal and left parietal alpha activity during NREM sleep cycles. This increase positively correlated with the amount of meditation training (Dentico et al., 2016). There is also evidence for increased frontal, parietal, and occipital alpha power and synchrony during meditation (Travis, 2001; Cahn et al., 2013). While both OM and FA have been shown to demonstrate an increased frontal alpha amplitude and synchrony (Travis, 2001), a recent study in awake patients demonstrated that the OM tradition (Vipassana) resulted in an increase in alpha power compared to meditation naïve controls and FA (Himalayan Yoga) practitioners during active meditation and mind wandering (Braboszcz et al., 2017). There is also evidence that experienced meditators have increased prefrontal and parietal alpha power during sleep (Dentico et al., 2016). There appears to be no consensus on the presence of parieto-occipital alpha in meditation practitioners, with some studies suggesting increased posterior alpha power, while other studies suggesting that FA and OM meditation decrease alpha (Dentico et al., 2016; Braboszcz et al., 2017).

There is evidence for increased alpha coherence in the frontal and parietal regions in the FA and OM traditions as well as TM (Travis, 2001; Cahn et al., 2013; Travis and Parim, 2017). In addition, as opposed to FA and OM, the predominant oscillation during TM is the frontal alpha rhythm as opposed to the theta rhythm. Transcendental Meditation practice has been associated with increased alpha power among the posterior cingulate gyrus, precuneus, and the medial and inferior temporal cortex (Travis and Parim, 2017; van Lutterveld et al., 2017).

## Beta frequency

Human beta oscillations (13–30 Hz) are typically associated with sensorimotor processing (Symons et al., 2016); however, more recently, they have been linked to attention, emotion, and cognitive control (Guntekin et al., 2013; Symons et al., 2016). There is conflicting evidence on the effects of meditation on beta oscillations. While there is some evidence that suggests no change in beta activity during OM (Pasquini et al., 2015), other studies suggest a decrease in beta activity in the angular gyrus and posterior cingulate and parietal cortices (Dor-Ziderman et al., 2013; Faber et al., 2015). There are also reports of decreased occipital beta oscillations during TM (Tomljenovic et al., 2016). Conversely, there is evidence that there is increased beta activity in the insula, inferior frontal gyrus and anterior temporal lobe during mindfulness meditation (Thomas et al., 2014; Schoenberg et al., 2017).

## Gamma frequency

Gamma oscillations in adult humans range between 30 and 100 Hz, and are thought to be involved in a number of sensory and cognitive responses (Pritchett et al., 2015; Kambara et al., 2017). In various forms of FA and OM traditions, practitioners have exhibited fast gamma activity with peak frequencies around 40 Hz in bilateral hemispheres seen only in highly advanced meditators (Fell et al., 2010). There is evidence of increased gamma activity in advanced practitioners of various meditation practices, including FA, OM, LK, and TM traditions. More specifically, in both FA (Himalayan Yoga) and OM practices (Vipassana, Isha and Shoonya Yoga), there is an increase in parieto-occipital gamma (60–110 Hz) compared to controls (Braboszcz et al., 2017). The posterior increase in gamma activity may be related to the often-described enhanced perceptual clarity reported in OM meditative processes (Cahn et al., 2010). In expert Tibetan Buddhist meditators, during compassion meditation there was a higher fronto-parietal gamma power (Lutz et al., 2004). Of note, one study suggested that during Zen meditation (another form of OM), high-frequency gamma power (100–245 Hz) in the cingulate cortex and somatosensory cortex positively correlated with the degree of self-reported mindfulness (Hauswald et al., 2015). While the functional role of gamma activity is not yet clear, one hypothesis is that it induces neuroplasticity via repetition, as it continues to be seen in more experienced meditators across different practices (Braboszcz et al., 2017). These studies suggest that mindfulness meditation practices increase gamma oscillations across multiple, but specific brain regions depending on the specific type of meditation.

In contrast to theta and alpha coherence, there is evidence for increased gamma oscillation coherence in the parieto-occipital regions during Vipassana and in the fronto-parietal regions in Buddhist practitioners (Lutz et al., 2004; Cahn et al., 2013). There is some evidence for gamma oscillation differences between the meditation traditions, as there is a reported increase in the gamma/alpha ratio in FA practitioners relative to OM practitioners (Braboszcz et al., 2017).

## Conclusion

Meditation results in significant changes in cortical and subcortical activity. As might be expected, different forms of meditation elicit activation of different regions of the brain. Previous work has demonstrated that electrographic oscillations are important in cognition. Here, we reviewed the effects of FA, OM, TM, and LK meditation on neural oscillatory activity.

Current research suggests that meditation has many beneficial effects on mood, consciousness, and awareness. Meditation results in structural and functional brain changes. During active meditation, there are distinct changes in the electrographic activity, both regionally and globally. There also appear to be distinct differences in EEG profiles depending on experience. One study on Satyananda Yoga practitioners demonstrated that intermediate (mean experience 4 years) practitioners had increased low frequency oscillations (theta and alpha) in the right superior frontal, right inferior frontal, and right anterior temporal lobes, whereas, advanced (mean experience 30 years) practitioners had increased high frequency oscillations (beta and gamma) in the same regions (Thomas et al., 2014). Advanced practitioners also seem to have more consistent electrographic changes and the concept of a meditative trait with neural oscillatory correlates is becoming clearer. While there have been correlations between electrical activity and behavior, further research needs to be performed to validate these correlations.

## Clinical implications and future directions

Determining the neural basis of meditation can potentially be used to improve meditation training and better understand neuronal circuitry. More specifically, oscillatory neurofeedback could be used to correlate an objective measure of brain activity with subjective experience, and thus be used as a tool for meditation training (van Lutterveld et al., 2017).

Understanding the interplay between meditation and the functional and anatomical correlates not only helps to inform how meditation benefits cognition, but it could potentially be used to determine targets for therapeutic neuromodulation. As many disease processes result in altered oscillatory patterns, identifying specific oscillatory aberrations in a disease state, and conversely meditation forms that reverse those traits, could lead to electrophysiological-based treatments. For example, the alpha and delta frequency bands are integral in sleep. Better elucidating the perturbations in neural oscillations could possibly be used to treat sleep disorders through meditation. Moreover, there are altered neural oscillations in neuropsychiatric disorders like depression, addiction, attention deficit hyperactive disorder, and bipolar disorder. In the future, meditation-based treatments could potentially be expanded for these neuropsychiatric disorders. However, as there are many types of meditation traditions, more rigorous studies need to be performed to elucidate the nuanced imaging and electrophysiological changes that occur with each type of meditation.

## Author contributions

DL, EK, PG, AG, and FG: participated in idea conceptualization, preparation, and editing of the manuscript.

### Conflict of interest statement

The authors declare that the research was conducted in the absence of any commercial or financial relationships that could be construed as a potential conflict of interest.
